# Our Choice: study protocol for a randomized controlled trial for optimal implementation of psoriasis treatment by the integration of Chinese and western medicine

**DOI:** 10.1186/s13063-020-4209-3

**Published:** 2020-03-30

**Authors:** Xiaoying Sun, Xiaoyong Zhou, Yuegang Wei, Wenxin Yang, Ning Huang, Yangfeng Ding, Rongyi Hu, Shun Guo, Chunyan Yang, Huilan Weng, Ying Zhang, Xi Chen, Xiaojie Ding, Liu Liu, Qingfeng Yin, Ruiping Wang, Xin Li, Bin Li

**Affiliations:** 1 Department of Dermatology, Yueyang Hospital of Integrated Traditional Chinese and Western Medicine, Shanghai University of Traditional Chinese Medicine, Shanghai, 200437 China; 2 Institute of Dermatology, Shanghai Academy of Traditional Chinese Medicine, Shanghai, 201203 China; 3 Department of Dermatology, Wuhan No. 1 Hospital, Wuhan, 430022 China; 4 Department of Dermatology, Affiliated Hospital of Nanjing University of Chinese Medicine, Nanjing, 210029 China; 5 Department of Dermatology, Traditional Chinese Medicine Hospital of Southwest Medical University, Luzhou, 646000 China; 6 Department of Dermatology, The Second Affiliated Hospital of Fujian Traditional Chinese Medical University, Fuzhou, 350001 China; 7 Department of Dermatology, Shanghai Dermatology Hospital, Shanghai, 200443 China; 8 Jiangsu Famous Medical Technology Co. Ltd., Nanjing University of Traditional Chinese Medicine, Floor 2, Building 19, Nanjing, 210029 China; 9 Office of Clinical Medical Research Center, Yueyang Hospital of Integrated Traditional Chinese and Western Medicine, Shanghai University of Traditional Chinese Medicine, Shanghai, 200437 China

**Keywords:** Plaque psoriasis, Integrated Chinese and western medicine, Jueyin granules, Moving cupping therapy, NB-UVB

## Abstract

**Background:**

Plaque psoriasis is a refractory inflammatory skin disease. The common therapies used to treat plaque psoriasis in traditional Chinese medicine (TCM) and western medicine (WM) have distinct characteristics and advantages. Although a combination of TCM and WM therapies, adjusted to the clinical situation, is widely used, there are no systematic studies on the hierarchical selection of this treatment combination based on the severity of skin lesions. We therefore designed a randomized clinical trial to focus on the sequence of internal and external treatments of TCM in patients with mild-to-moderate plaque psoriasis and to optimize the integration of Chinese and western medicine for the treatment of patients with severe plaque psoriasis, thereby achieving high-level clinical evidence and establish treatment norms for the integrated use of Chinese and western medicines.

**Methods:**

In this proposed multicenter, single-blinded, randomized controlled trial, 108 patients with mild-to-moderate plaque psoriasis will be randomly assigned to two groups in a 1:1 ratio to receive either internal or external TCM treatment, and 270 patients with severe plaque psoriasis will be randomly assigned to three groups in a 1:1:1 ratio to receive treatment with TCM or WM, or TCM + WM. All enrolled patients will receive 8 weeks of treatment. Follow-up assessments will be done 8 weeks after the treatment. The primary outcome of this study is the evaluation of efficacy and relapse rate, based on the Psoriasis Area and Severity Index, and the secondary outcome measures include determination of the affected body surface area, physician’s global assessment, pruritus scores (determined using a visual analog scale), TCM symptom score, Dermatology Life Quality Index, patient-reported quality of life score and incidence of serious adverse events.

**Discussion:**

This study will provide high-level clinical evidence for internal and external TCM treatment optimization and will contribute to establishing norms for the integration of Chinese and western Medicines.

**Trial registration:**

ClinicalTrials.gov, NCT03941431. Registered on 8 May 2019.

## Introduction

### Background and rationale

Psoriasis is a common chronic relapsing inflammatory skin disease with a prevalence rate of 2–4% [1]. Although its underlying cause is not fully understood, it is generally associated with genetic, metabolic, immunological, endocrinal and infective etiologies [2]. Psoriasis, particularly refractory plaque psoriasis, can have considerable detrimental effects on the patient’s quality of life. In western medicine (WM), treatments for psoriasis, such as topical corticosteroids, vitamin D derivatives, calcineurin inhibitors, systemic phototherapy, acitretin, cyclosporine A, immunosuppressants and biological agents [3], can alleviate the clinical symptoms to varying degrees. However, the potential safety problems and high costs associated with the use of these agents often limit their clinical application.

The treatment of psoriasis using traditional Chinese medicine (TCM) has a long history, with the earliest records traced back to more than 1400 years ago. It is based on a completely theoretical system, which has evolved progressively in response to technological developments and lifestyle changes. A series of systematic reviews of TCM in clinical practice have indicated that this approach is effective in the treatment of psoriasis [4–10], and clinical and experimental data indicate that TCM can modify psoriasis by antagonizing or regulating interleukin (IL) and the IL-23/IL-17 axis to inhibit the main causal pathways [11].

In a clinical trial conducted by our research team, we found that treatment with Jueyin (JY, 决银, 決銀) prescription, a compound Chinese herbal preparation containing seven constituents (abalone shell, honeysuckle, tree peony bark, dried rehmannia root, *Hedyotis diffusa*, folium and turmeric root tuber; Table 1) is safe and effective in patients with early-stage psoriasis [12]. Its mechanism of action is related to the inhibition of keratinocyte proliferation, enhancement of epidermal parakeratosis, and reduction in nitric oxide and malondialdehyde [13]. These assumptions have accordingly been verified in in vitro studies, in which a 5% JY prescription had a significant inhibitory effect on the proliferation of HaCaT cells, with primary effects on the G1 phase of the cell cycle [14].

**Table 1:** Constituents of the Jueyin prescription

Main constituents	Scientific name	Plant part(s)
Abalone shell	*Concha haliotidis*	Shell
Honeysuckle	*Lonicera japonica* Thunb	Flower
Tree peony bark	*Cortex Moutan*	Bark
Dried rehmannia root	*Rehmannia glutinosa* Libosch	Root
Hedyotis diffusa	*Hedyotis diffusa* Willd	Whole grass
Folium	*Isatidis*	Leaf
Turmeric root tuber	*Curcuma aromatica* Salisb	Root

In TCM, the moving cupping therapy is a type of acupuncture therapy, commonly used in China and other Asian countries, and is gradually gaining worldwide acceptance owing to its simplicity, convenience and effectiveness [15]. Compared to treatment with oral Chinese medicine alone, a more pronounced decrease in Psoriasis Area and Severity Index (PASI) score has been recorded in psoriasis patients treated with moving cupping therapy combined with TCM [16]. In this procedure, a vacuum is generated by heating air in a cup with a flame and placing the cup over skin lesions. The cupping produces a mild attraction in the skin, which is characterized by a strong adsorptive force and a deep action on skin lesions. Coupled with the rapid push and pull of the cup against the body at the site of the skin damage, cupping accelerates blood circulation and promotes metabolism, dissipates blood stasis, regulates meridians and collaterals, and stimulates the overall vitality of the body to strengthen its resistance and eliminate pathogenic factors. Previous studies have shown that the moving cupping therapy combined with TCM ointment is effective in the treatment of plaque psoriasis, having an effect equivalent to the exposure to narrow-band ultraviolet B radiation (NB-UVB) combined with externally applied calcipotriol ointment [17].

NB-UVB phototherapy is a common and effective method for the treatment of plaque psoriasis. An optimal wavelength of 313 nm of NB-UVB phototherapy has been shown to have high efficacy and a small side-effect profile [18, 19]. It has been reported that UV light affects various components of the natural and acquired immune responses and is related to the depletion of both the Langerhans cells and T cells in the epidermis [20–22]. Previous studies have indicated that, in patients with psoriasis, the serum levels of 25-hydroxyvitamin D increase in response to NB-UVB treatment, and there is a correlation between this increase and the number of therapy sessions [23]. Moreover, changes in the skin microflora following UVB treatment may be related to treatment response [24]. These findings thus indicate that the effectiveness and safety of phototherapy on psoriasis may depend on a complex interaction of immunological and metabolic mechanisms. In addition, according to clinical reports, NB-UVB combined with a TCM-medicated bath can improve the curative effect, reduce the cumulative dose, and lessen the adverse reactions of UV radiation compared to exposure to NB-UVB alone [25].

### Objectives

Although TCM and WM are both commonly used in the treatment of psoriasis and each has its own associated advantages and characteristics, the combined application of these two approaches can enhance the curative effect and reduce the side effects and recurrence rate of psoriasis. Therefore, the combination of TCM and WM therapies, adjusted according to the clinical situation, has been used widely in medical practice. However, there have been no previous clinical studies examining the hierarchical selection of treatment combinations according to the severity of skin lesions. To date, only the pairwise combinations of Chinese herbal medicine, cupping therapy and NB-UVB phototherapy for plaque psoriasis have been reported, and the curative effects were found to vary. Therefore, in this multicenter, randomized controlled, single-blind clinical trial, “Our Choice”, we combined JY granules (JYG) and moving cupping therapy for the treatment of mild-to-moderate plaque psoriasis, while for severe psoriasis we combined JYG, moving cupping therapy and NB-UVB phototherapy. This study aimed to optimize the internal and external TCM treatments and establish a high level of clinical evidence and treatment norms for the integration of Chinese and western medicines.

### Trial design

This is a multicenter, single-blinded, randomized controlled trial that aims to determine the appropriate time for the intervention with TCM and involves a sequential treatment plan for severe psoriasis by combining TCM and WM.

The study consists of three phases: screening, treatment and follow-up. During the initial screening period, patients will be recruited to dermatological clinics for body surface area (BSA) assessments and laboratory tests (including pregnancy tests for women of child-bearing age), and they will also be assessed on the designated inclusion and exclusion criteria. The eligible patients will be required to sign a written informed consent form, including an option to participate in the biobank initiative. The investigator will clearly explain to the patients all the details in the consent form, ensuring that they are fully aware of their rights and are able to cooperate with the researchers to complete both the treatment and follow-up phases. Patients who provide their informed consent and meet the laboratory test criteria will undergo the medical procedures, based on their BSA score. According to the latest Chinese guidelines for severity, psoriasis is classified into the following categories: mild (lesion area <3% BSA), moderate (lesion area 3–10% BSA) and severe (lesion area >10% BSA) [26]. In this study, only those patients with skin lesions involving 10–15% BSA will be included in the severe group.

**Fig. 1:**
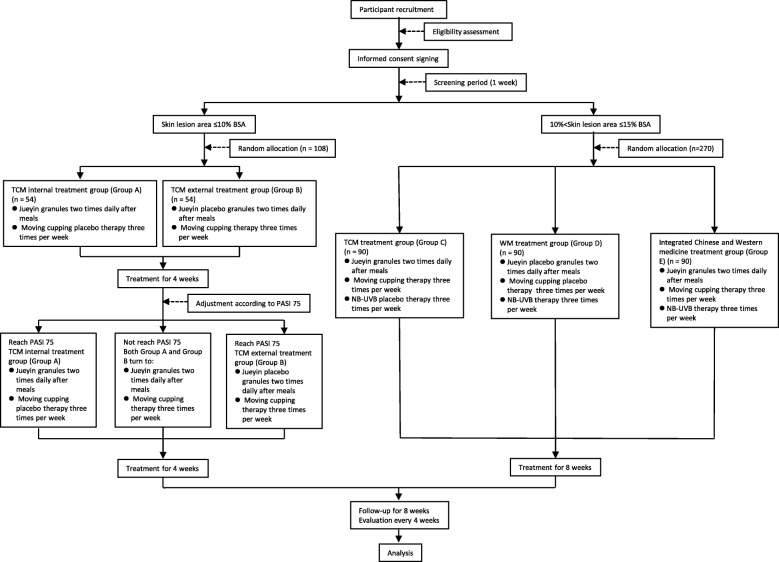
Study flowchart. BSA body surface area, NB-UVB narrow-band ultraviolet B radiation, PASI 75 75% reduction in the Psoriasis Area and Severity Index, TCM traditional Chinese medicine, WM western medicine

Patients with mild-to-moderate plaque psoriasis will be randomly assigned to one of two groups in a 1:1 ratio, where one group will receive JYG and moving cupping placebo therapy and the other group will receive JYG placebo (JYGP) and moving cupping therapy. The patients in these two groups will be evaluated after 4 weeks of treatment. If a 75% reduction in the PASI (PASI 75) is achieved, treatment will be continued until the end of the 8-week study. Otherwise, patients receiving the moving cupping placebo therapy will be switched to moving cupping therapy, and those receiving JYGP will be switched to JYG for the subsequent 4 weeks of treatment. Patients with severe plaque psoriasis will be randomly assigned to one of three groups in a 1:1:1 ratio to receive JYG, moving cupping therapy and NB-UVB placebo therapy, or JYGP, moving cupping placebo therapy and NB-UVB therapy, or JYG, moving cupping therapy and NB-UVB therapy (Fig. 1). At each visit, we will evaluate and record the relevant patient efficacy indicators and TCM symptom information and obtain images of target lesions. This manuscript has been written in accordance with the Standard Protocol Items: Recommendations for Interventional Trials (SPIRIT) 2013 checklist (see Additional file 1). The results of the study will be disseminated to the public through conference reports and open-access journals.

## Methods: participants, interventions and outcomes

### Study setting

The study will be conducted at the following six centers in China: the Shanghai Yueyang Integrated Medicine Hospital, the Shanghai Dermatology Hospital, the Chinese Medicine Hospital Affiliated to the Southwest Medical University, the Wuhan No. 1 Hospital, the Second Affiliated Hospital of Fujian Traditional Chinese Medical University and the Affiliated Hospital of the Nanjing University of Chinese Medicine. Patients will be assessed in the dermatology departments of each hospital. Each study center has a solid work foundation and sufficient outpatient volume, which provides supporting conditions for the completion of this study.

### Eligibility criteria

#### Inclusion criteria

To be eligible for recruitment, the patient must:

1. Have plaque psoriasis; the course of the disease is not limited
2. Have skin lesions covering ≤15% of the BSA (it should be mainly located on the trunk and/or limbs, palms/soles and face/scalp; the vulva area should not be included)
3. Aged between 18 and 65 years
4. Provide informed consent

#### Exclusion criteria

Patients who meet any of the following criteria will not be eligible for recruitment:

1. Patients with other active skin diseases that may affect condition assessment
2. Patients who have received systematic treatment with research drugs, biological agents or immunosuppressive agents within 2 months prior to screening
3. Patients who have received treatment with topical glucocorticoids, retinoic acid or phototherapy within 2 weeks prior to screening
4. Patients with severe, uncontrollable local or systemic acute or chronic infections
5. Patients with severe systemic diseases or any of the following clinical test indicators: an increase in alanine transferase or glutamate transferase level >1.5 times the upper limit of normal or an increase in serum creatinine level >1.5 times the upper limit of normal; patients with any of the main standard blood indicators (i.e., white blood cell count, red blood cell count, hemoglobin level and platelet count) below the lower limit of normal, or those with other laboratory abnormalities as judged by the investigators, will be excluded
6. Patients with a history of malignant tumors or primary/secondary immunodeficiency and hypersensitivity
7. Patients who have undergone a major surgery within 8 weeks of treatment or will require such surgery during the study period
8. Patients who are pregnant or lactating
9. Patients with a history of alcohol or drug abuse
10. Patients with a history or family history of a serious mental illness
11. Patients with a family history of cancer
12. Patients who were judged by the investigators to be unsuitable for inclusion in the study for any other reasons

### Who will take informed consent?

All patients will receive professional and practical knowledge of their daily treatment and nursing from a team of researchers, including senior experts in the field of TCM for psoriasis and will be provided with feedback on the results of the relevant assessment on completion of the study. When signing the informed consent form, the patients will be informed of the burden of the study and their freedom to withdraw from the study at any time. In case patients decide to withdraw from the study, they will be required to undergo a final evaluation of efficacy and safety.

### Interventions

#### Intervention description

Patients in the TCM internal treatment group (group A) will receive JYG twice daily after meals and moving cupping placebo therapy three times weekly for 8 weeks, while patients in the TCM external treatment group (group B) will receive JYGP twice daily after meals and moving cupping therapy three times weekly for 8 weeks. Patients in the TCM treatment group (group C) will receive JYG twice daily after meals and moving cupping therapy and NB-UVB placebo therapy three times weekly for 8 weeks, while those in the WM treatment group (group D) will receive JYGP twice daily after meals and moving cupping placebo therapy and NB-UVB therapy three times weekly for 8 weeks. In the TCM + WM treatment group (group E), the patients will receive JYG twice daily after meals and moving cupping therapy and NB-UVB therapy three times weekly for 8 weeks.

#### Moving cupping therapy

According to the body position requirements during the moving cupping therapy, the patient will be asked to take a suitable sitting or lying position. Subsequently, the operator will expose the local skin, select the acupoint location, and apply a small amount of Vaseline oil to lubricate the skin after routine disinfection. Cups of appropriate size with smooth mouths will be used. The operation will start after disinfection. The operator will adhere to the following procedure during the operation: using the left hand to hold a 95% alcohol cotton ball, the operator will ignite the middle and lower sections of the cup. The operator will then take the cup out after moving it in a circle. Next, holding the cup in his right hand, he will quickly place it at the concentrated site of the lesion, pushing it toward the distal end and turning back. These steps will be repeated several times. Patients will be required to wear an eye patch during the entire operation. Slow walking refers to moving the cupping back and forth approximately once per second, whereas fast walking refers to moving the cupping back and forth approximately twice per second. According to the clinical requirements, a combination of light adsorption and slow walking, light adsorption and fast walking, heavy adsorption and slow walking, and heavy adsorption and fast walking should be chosen. The combination of heavy adsorption and fast walking is often used in the lower back or limbs of patients with plaque psoriasis. The cup will be replaced every five times, with an interval of no more than 10 s. Each part of the moving cupping will be positioned on the patient’s skin about 30 times until the local skin appears reddish, red, crimson or purplish.

#### Moving cupping placebo therapy

**Fig. 2:**
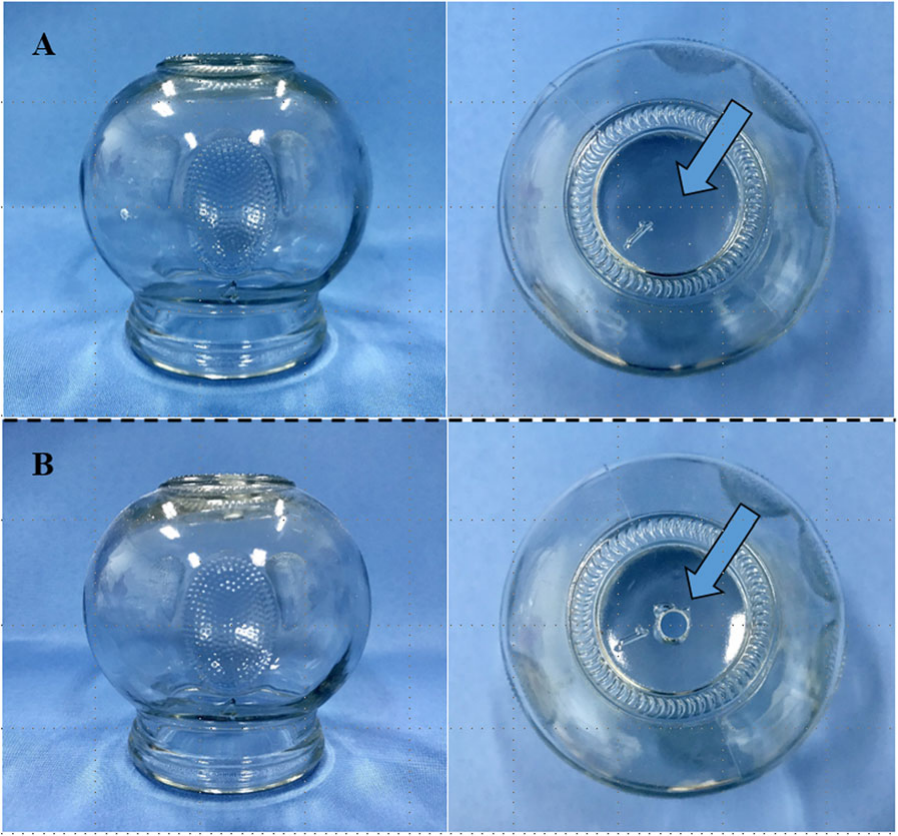
Cups used in the moving cupping therapy and placebo therapy. **a** Cups used in the moving cupping therapy. **b** Cups used for moving cupping placebo therapy

The procedure for moving cupping placebo therapy will be similar to the moving cupping therapy. In this operation, the same glass cup will be used except that it will have 6 mm holes drilled in the top. Patients undergoing the moving cupping placebo operation will also need to wear an eye patch during the procedure. After the oxygen combustion in the cup is consumed, the air can enter through the small hole to supplement it; therefore, the negative pressure adsorption force will not be formed. This simulates moving cupping therapy without the therapeutic effect and is similar to mild scraping therapy, leaving a similar reddish mark on the skin (Fig. 2).

#### Strategies to improve adherence to interventions

All patients in each of the treatment groups will be provided with a free of charge emollient (YuZe Skin Barrier Recovery Body Lotion®; developed by the Rui Jin Hospital and produced by Shanghai Jahwa United Company, China) as basic skin care. We anticipate that this will enhance patient compliance. To document compliance, patients will be required to record the dose of the oral granules and the time of administration in a daily diary of medication. At each visit, the researchers will record the application dates and doses of emollients, drugs, moving cupping therapy and NB-UVB phototherapy. Unused emollients and drugs will be returned to the hospital prior to the distribution of new emollients and drugs.

In the preparatory phase of the trial we fully considered the time cost of each follow-up. In the debugging phase of the case management system we added the mobile telephone upload function of the patient laboratory examination report and skin lesion photos and set up the treatment follow-up reminder function to reduce the loss of visit rate. The patients who do not follow-up on time will receive repeated reminders from researchers in the time window.

### Outcomes

#### Primary outcome

The primary outcome in this trial is evaluation of the efficacy and incidence of relapse in response to sequential treatment with integrated Chinese and western medicines during the treatment and follow-up periods. Relapse is defined as a PASI score exceeding the baseline score at the time of enrollment or development of new pustules or erythroderma [27]. PASI will be assessed at baseline, at 2-week intervals during the treatment period, and at 4-week intervals throughout the follow-up period.

#### Secondary outcomes

The secondary outcome measures in the study are: 1) improvement in BSA; 2) improvement in physician’s global assessment (PGA); 3) visual analog scale (VAS) scores for pruritus; 4) improvement in TCM symptom score; 5) improvement in the Dermatology Life Quality Index (DLQI) and patient-reported quality of life (PRQoL); and 6) incidence of serious adverse events (SAEs).

The BSA, PGA, VAS score, TCM symptom score, DLQI and PRQoL will be assessed at baseline, at 2-week intervals during the treatment period, and at the end of the follow-up period. Laboratory tests, including complete blood counts, urinalysis, and hepatic and renal function tests, will be performed at baseline and at 8 weeks after treatment (Table 2).

**Table 2:**
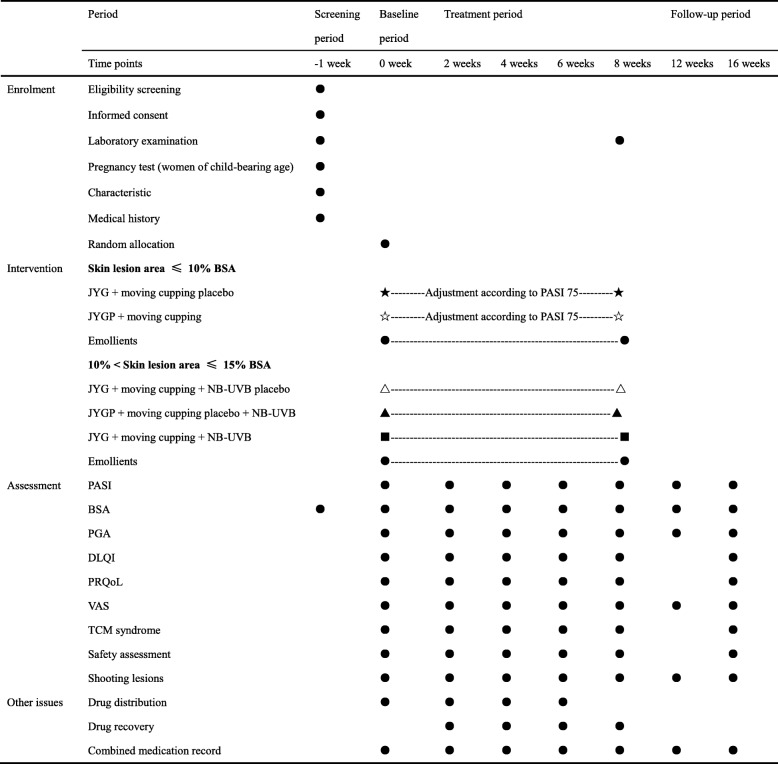
Schedule for treatment and outcome measurements

*BSA* body surface area, *DLQI* Dermatology Life Quality Index, *JYG* Jueyin granules, *JYGP* Jueyin granules placebo, *NB-UVB* narrow-band ultraviolet B radiation, *PASI* Psoriasis Area and Severity Index, *PASI 75* 75% reduction in the Psoriasis Area and Severity Index, *PGA* Physician Global Assessment, *PRQoL* patient-reported quality of life, *VAS* visual analog scale, *TCM* traditional Chinese medicine

### Sample size

The sample size for this study was calculated based on the expected value of the efficacy based on the results from previous studies [17, 28]. The efficacy rates for groups A, B, C, D and E were 30.0%, 59.0%, 73.0%, 55% and 88.0%, respectively. The significance level (alpha) was set at 0.05 and the statistical power was 80%. Based on our calculation using the PASW statistical software (V.18.0), a sample size of 43 patients was required for groups A and B and 72 patients for groups C, D and E. However, considering a potential 25% loss of patients during follow-up, we adjusted the total sample sizes of groups A and B to 54 patients and that of groups C, D and E to 90 patients.

### Assignment of interventions: allocation

#### Sequence generation

Eligible patients with mild-to-moderate or severe psoriasis will be enrolled at each of the six participating institutions and will be randomized separately. During their second visit, patients with mild-to-moderate psoriasis will be randomly assigned to either group A or group B in a 1:1 ratio, while those with severe psoriasis will be randomly assigned to group C, group D or group E in a 1:1:1 ratio. Randomization will be performed using a computer-generated random assignment sequence through the central layering and block randomization method of SAS software (V.9.4).

#### Concealment mechanism

A unit unrelated to this clinical trial will be designated to package and distribute the drugs (test and control) under the supervision of statisticians. The allocation concealment will be ensured. A randomization code will be released using a data network platform designed by the data management center of Jiangsu Famaisheng Medical Technology, and the random code will correspond to the drug number and external treatment group. Thereafter, the patients will be allocated to the different treatment groups under randomization concealment.

The JYG and corresponding placebo granules that will be used in this trial were prepared by Sanjiu Pharmaceuticals (Hefei, Anhui Province, China). These drugs meet the requirements of Good Manufacturing Practice for Pharmaceutical Products. The placebo granules are similar to the JYG constituents in terms of appearance, weight and taste, with the main constituents being maltodextrin, lactose and a natural edible pigment.

The glass cups that will be used for the moving cupping therapy were obtained from Guandong Glass Products (Haimen City, Jiangsu Province, China). The moving cupping placebo therapy will be performed by drilling a hole on the top of the tank (0.6 cm in diameter). The moving cupping placebo therapy will have a push-and-pull effect on and around the skin lesion but will not produce a negative pressure attraction effect. The standard operating procedure of moving cupping will be in accordance with Part 5, Cupping of the People’s Republic of China Standard G/B21709.5–2008. In both moving cupping therapy and its placebo, we will use white Vaseline as a lubricating matrix. NB-UVB therapy will be performed using an NB UV wave therapy device, and the placebo therapy will be performed by adjusting the dose to 100 mJ/cm^2^. The moving cupping therapy and phototherapy will be performed by trained researchers, and patients will be required to wear an eye patch while the treatment is being performed. Patients in all five treatment groups will undergo similar medical procedures and will be blinded to the allocation. Additionally, it should be noted that the fortnightly clinical evaluation during the treatment period will be evaluated before the moving cupping therapy to ensure that the investigators are blind to the group allocation.

### Data management

All researchers and research assistants will attend training seminars before the start of the trial. Researchers at different centers will be requested to follow the same standard operating procedures. Data entry will be completed using the case management system specifically designed for this trial by Jiangsu Famaisheng Medical Technology. To ensure quality and consistency between the source data and the data entered into the system, the data will be entered separately at each center by two research assistants and reviewed by the lead investigator after the completion of each visit. The quality control personnel of the Shanghai Yueyang Integrated Hospital (Shanghai, China) will regularly monitor the data collected at each participating center during the entire study period. The final data will be reviewed and inspected by the Office of State Key Technology R&D Program of the Ministry of Science and Technology of China.

### Statistical methods

All analyses will be performed by applying the SPSS V.21.0, (SPSS Inc., Chicago, IL, USA) statistical software and the data network platform designed by the data management center of Jiangsu Famaisheng Medical Technology. The outcome investigators will be blinded to group allocation. According to the principle of an intention-to-treat analysis, all patients who will be randomized and evaluated according to the efficacy indicators at least once are defined as the full analysis set population, whereas all patients who meet all inclusion and exclusion criteria and deviated from the prescribed dose less than 20% of the regimen are defined as the per-protocol set population. Patients who received treatment one or more times and have at least one safety evaluation will be analyzed in the safety set population. The efficacy measurement will be evaluated mainly by intention-to-treat analysis (full analysis set) and supplemented by per-protocol analysis. The safety evaluation will be analyzed in the safety set population.

If a numerical variable shows normal distribution and the variance is uniform, the statistical description will be expressed as the mean ± standard deviation; the statistical inference will be expressed by the F and S-N-K tests; and the repeated measurement of data analysis of variance will be used for comparison among the groups at multiple time points. In case a numerical variable shows skewed distribution, the median and minimum and maximum values will be used for statistical description; the rank sum test and Nemenyi test will be used for statistical inference, and the generalized estimation equation will be used for comparison among the groups at multiple time points. For categorical variables, the frequency, constituent ratio and rate will be used for statistical description. If the analysis index is unordered, a chi-squared test will be used for statistical inference and, if the analysis index is ordinal, a nonparametric test will be used for statistical inference. The generalized estimation equation will be used for comparison among the groups at multiple time points. Hypothesis testing will be performed using a two-sided test with 95% confidence intervals. A *P* value less than 0.05 indicates statistical significance.

### Adverse event reporting and harms

Medical history records will be prepared for each patient, which will include the results of the standard laboratory examinations before the start of the study and after 8 weeks of treatment. Standard laboratory examinations will include the following: routine blood tests, routine urine tests and indices of renal function (uric acid, creatinine and urea) and hepatic function (alanine aminotransferase, aspartate aminotransferase, total bilirubin and γ-glutamyl-transpeptidase).

At each of the 2-week visits during treatment, the investigator will collect data pertaining to all adverse events (AEs), their severity, and their potential relationship to treatment. The safety assessment will include the determination of the incidences of treatment-related adverse events (trAEs) or SAEs, the rate of trAEs contributing to discontinuation, and changes in laboratory parameters. If SAEs occur, all medications and therapies in this trial will be discontinued immediately.

Any treatment and results of AEs during the study should be recorded in the AE list. Researchers should decide the diagnosis, treatment measures, and follow-up forms according to the severity of AEs, such as hospitalization, outpatient service, home visit, telephone, and so forth, until the laboratory index returns to normal and the symptoms and signs disappear. The cost of handling all trAEs should be borne by the unit responsible. In case of SAEs, the subcenter will take immediate measures necessary to protect the safety of the patients.

## Discussion

Psoriasis is an immune-related disease characterized by a gradual long-term development and frequent recurrence of symptoms; it has been shown to negatively affect the quality of the life in adults and children due to social isolation, occupational stress and stigmatization [29–36]. On the basis of an in-depth survey of the recent clinical trials and research on the underlying mechanisms, we found that psoriasis is a chronic multisystem inflammatory disease associated with a variety of related complications, including hypertension, cardiovascular disease, metabolic syndrome, malignant tumors and inflammatory bowel disease [37, 38]. Moreover, psoriasis has emerged as a condition that is indicative of an increased risk of disease and death associated with these complications [39].

Three types of psoriasis syndrome are widely recognized based on TCM theory, namely blood heat, blood stasis and blood dryness. Blood heat is considered a key pathological factor in the progression of psoriasis, while blood dryness is mostly caused by long-term psoriasis symptoms. Blood stasis may be present throughout the course of the disease, especially in plaque psoriasis. TCM is widely applied as the first-line treatment for mild-to-moderate psoriasis due to its suitability for long-term use at reasonable compatibility and doses [40]. JY is a representative prescription for the blood-heat syndrome of psoriasis and in the treatment of plaque psoriasis. It consists of seven constituents that are involved in clearing of heat, cooling of blood, and detoxifying and removal of blood stasis.

In a previous study, we have found that JY prescription was an effective and safe medication for mild-to-moderate psoriasis vulgaris, with no obvious adverse reactions [12]. In addition to mechanistic research, we have also conducted acute and chronic toxicity tests for 6 months in a mouse model of psoriasis and found that treatment with JYG did not result in any significant abnormalities in the physiological parameters or pathological changes in the major organs of rats [41]. We therefore considered JYG as a safe and effective medication for the treatment of psoriasis. Results from a pooled analysis conducted by us also indicated that levels of interferon-γ, IL-17, IL-23, and tumor necrosis factor-α were significantly increased, whereas those of IL-4 and IL-10 were significantly decreased in the sera of patients with blood-heat syndrome of psoriasis [42]. Determining the effect of the JY prescription on the aforementioned immune factors will be a focus area of our future research. To ensure the high treatment compliance in the present trial, we intend to use JY prescription granules instead of Chinese herbal medicine, owing to the establishment of its quality control standard using high-performance liquid chromatography [43].

Many clinical trials in China have shown that the combination of oral TCM and external treatment for psoriasis show superior clinical effects compared to individual TCM treatment or external treatment. The moving cupping therapy is particularly effective for the treatment of plaque psoriasis because it is noninvasive with no side effects and is therapeutically advantageous, simple, convenient and inexpensive. Furthermore, it promotes lesion thinning and regression. Considering that the efficacy of moving cupping therapy in the 8-week treatment of plaque psoriasis was higher than that in the 4-week treatment [44], we designed the entire treatment cycle to run for 8 weeks.

In the present study, patients with mild-to-moderate psoriasis will be treated with TCM. After 4 weeks of treatment, depending on whether PASI 75 is reached, the necessity to adjust treatment from JYGP to JYG or the moving cupping placebo therapy to moving cupping therapy will be evaluated. This novel study design facilitates not only a comparison of the curative effects of internal and external TCM treatments, but also enables an assessment of the intervention opportunities for internal and external treatment with TCM. The use of sequential treatment of TCM is in line with clinical practice.

For the purpose of the present trial, we intend to conduct NB-UVB phototherapy three times weekly because additional exposure and higher UVB doses can increase the incidence of adverse reactions, although for some patients a frequency of five exposures a week is associated with a more rapid cure of psoriasis [45, 46]. Moreover, NB-UVB phototherapy three times a week can be combined with moving cupping therapy to enhance patient compliance. Patients with severe plaque psoriasis will be treated separately with TCM, WM or TCM + WM to achieve rapid and long-term effects with no side effects and to establish high-level clinical evidence and treatment norms for integrated Chinese and western medicine.

In addition, the use of a moisturizer is necessary in any course of treatment for plaque psoriasis. Therefore, all the patients in this study will receive a basic skin care product (YuZe Skin Barrier Recovery Body Lotion®, a moisturizer containing linoleic acid-ceramide). Clinical trials have shown that local use of this moisturizer can relieve psoriasis and may be an effective approach in its treatment and prevention [47].

According to the 2018 guidelines for the diagnosis and treatment of psoriasis, TCM is the first choice of treatment for mild-to-moderate psoriasis, while integrated traditional Chinese and western medicine is needed for the treatment of severe psoriasis [26]. In this trial, we will compare TCM granules, moving cupping therapy, and NB-UVB phototherapy with placebo granules or placebo therapy to obtain a high-grade clinical evidence for the clinical application of this guideline. There is no stratification based on TCM syndromes because JYG can clear heat, cool blood and detoxify and remove blood stasis. A large cohort study on the syndrome differentiation and treatment of TCM for psoriasis vulgaris has been carried out in another subtopic of the National Key Research and Development Program of China [48]. As an important part of the research project, this study will contribute to determining the basic principles and application of the “new blood syndrome theory” and promote the innovation of the academic connotation of “TCM differentiation of blood treatment theory” for psoriasis. The results will provide a new perspective on the timing and options for therapy, based on internal and external TCM treatments and integrated Chinese and western medicine treatments for plaque psoriasis.

Our study has a few limitations, explained as follows. First, although all results will be measured and recorded by independent researchers to minimize the risk of detection bias, we cannot use a double-blind procedure in the moving cupping therapy and NB-UVB phototherapy. Second, small deviations in the manipulation of operators implementing moving cupping therapy are inevitable, but performance bias can be reduced through unified professional training. At present, a research team is developing negative pressure-controllable moving cupping appliances, which are expected to be used in future clinical trials.

## Trial status

This is protocol version 3.0 (3 May 2019). Patient recruitment began in September 2019 and is expected to be completed by the end of September 2021. The study procedures are expected to be completed by the end of December 2021.

## Supplementary information

**Additional file 1.** Standard Protocol Items: Recommendations for Interventional Trials (SPIRIT) 2013 checklist: recommended items to address in a clinical trial protocol and related documents.

## Data Availability

Not applicable.

## Abbreviations

TCM: Traditional Chinese medicine
WM: Western medicine
JY: Jueyin
PASI: Psoriasis Area and Severity Index
NB-UVB: Narrow-band ultraviolet B radiation
JYG: Jueyin granules
BSA: Body surface area
JYGP: Jueyin granules placebo
PASI 75: 75% reduction in the Psoriasis Area and Severity Index
PGA: Physician’s global assessment
VAS: Visual analog scale
DLQI: Dermatology Life Quality Index
PRQoL: Patient-reported quality of life
AE: Adverse event
SAE: Serious adverse event
trAE: Treatment-related adverse event
IL: Interleukin
